# The Effects of Social Identity and Emotional Connection on Subjective Well-Being in Times of the COVID-19 Pandemic for a Spanish Sample

**DOI:** 10.3390/ijerph181910525

**Published:** 2021-10-07

**Authors:** Jesús M. Canto, Macarena Vallejo-Martín

**Affiliations:** Department of Social Psychology, Social Work, Social Anthropology and EAS, Faculty of Psychology and Speech Therapy, University of Malaga, 29071 Málaga, Spain; mvallejo@uma.es

**Keywords:** social identity, emotional connection, social cure, subjective well-being

## Abstract

This study analyzes whether the degree of social identity and the degree of emotional connection influence the subjective well-being of individuals that participated in collective acts of support for health personnel fighting against the COVID-19 pandemic. Our sample was composed of 810 participants who resided in Spain (339 women and 471 men) with an average age of 34.22 (SD = 12.56). All of them frequently participated in the acts of support that took place each day of the lockdown decreed by the National Government on 14 March 2020. The results show that the greater identification with the group (the country) and the greater the emotional connection, the higher the scores obtained in subjective well-being. The results also show that emotional connection had a positive effect on emotional subjective well-being, mediated by the social identity activated in the collective act. The results are interpreted from the perspective of social identity that highlights the role played by social identity in influencing health and subjective well-being.

## 1. Introduction

From December 2019 to the end of December 2020, more than 82 million people throughout the world have been infected by COVID-19, with more than 1.8 million deaths. In the global context, the United States is the most affected country, with 19.7 million infections and 348,000 deaths, followed by Brazil and India [1]. Within the context of the European Union, Spain is nearing the two million-infection mark and has seen more than 50,000 deaths, ranking third in the world in number of deaths from the coronavirus per inhabitant, behind Belgium and Peru [2]. Because of the spread of the SARS-CoV-2, more than half of the world’s population has been subjected to some type of confinement, social distancing has been imposed and citizens’ movement and mobility have been restricted, which in addition to the health crisis, has brought about a grave economic and social crisis [3].

On 30 January 2020, the World Health Organization (WHO) declared that the outbreak of COVID-19 represented a public health emergency of international concern. On 11 March 2020, WHO officially declared the coronavirus pandemic [4]. In response to the pandemic, the Spanish Government declared a state of emergency on March 14, which made it possible to order confinement of the population. The Spanish Government ended this lockdown on June 21, after 99 days of confinement. In the wake of the second wave provoked by expansion of the coronavirus after the summer months, this same Government again declared a state of emergency on October 25 in order to establish less restrictive confinement measures, administered this time by the governments of Spain’s different autonomous communities.

Unlike other catastrophes that tend to be of shorter duration and whose impacts are located within a specific physical space (for example, earthquakes or bombings), the effects of the COVID-19 pandemic are prolonged and affect the economy of the different countries [5]. As it is a situation of risk, uncertainty and tension in terms of health as well as the economy, the current pandemic has triggered an array of negative emotions and behavior in a large part of the population associated with the risk of contracting the illness and the consequences of the economic crisis [6]. Even though the complex situation brought about by COVID-19 has led to a perception of government officials as leaders who are not always effective, the immense majority of the population obeyed the orders decreed by governments regarding compliance with the restrictions on behavior that entailed establishment of the different lockdown measures in the spring of 2020 [7]. Among the possible explanations for incompliance with instructions and orders from the authorities, we can point to mistrust on the part of some of the population toward governmental agencies along with confusion stirred up by information coming from different political and health authorities [8]. In this respect, the data from a study carried out in Australia, in the wake of the SARS-CoV-2 coronavirus pandemic, revealed that individuals that trusted the Government and other officials were more likely to follow recommendations on hygiene practices and limit their social interactions to avoid the spread of infection [9].

### 1.1. Social Identity, Emotional Connection and Subjective Well-Being

In a crisis such as that brought about by the pandemic, people face the same threat and share the same experience, which leads to a sense of a common destiny that entails a significant awareness of a shared social identity [5]. The main objective of this paper is to analyze if the level of social identity and the emotional connection that participants in the study had with their country during moments of the COVID-19 pandemic had any impact on their subjective well-being. Diener et al. [10] defined the subjective well-being as a person’s cognitive and affective evaluations of his or her life. The cognitive element refers to what a person thinks of his or her life satisfaction in global terms and in specific areas of his or her life. Further, the affective element refers to emotions, moods, and feelings, which can be positive (when emotions, moods and feelings experienced are pleasant) or negative (when they are not pleasant). Today there is an emerging research agenda that shows that social connections grounded in shared group membership and social identities have beneficial effects on health and well-being [11,12]. The perspective of social identity on group processes [13] assume that “individuals can self-define in terms of their own unique individual traits and features (personal identities) or in terms of their social identity (the group memberships they share with others; [14]). When someone defines himself or herself in terms of personal identity, he focusses on perceiving himself as an individual who is different from other individuals [15]. However, when defined through social identify, individuals focus on the similarities that they have with others with whom they share this social identity (ingroup members) and on their difference from those with whom they do not share group membership (outgroup members). Sharing group memberships impacts on the psychology of individuals (including their health and well-being) through their capacity to be psychologically internalized as part of the self [16]. When we think about ourselves and about others as members of the same group sharing the same social identity, it entails important psychological consequences [13]. Specifically, it results in increased trust, respect and communication among members of the ingroup [17], greater intragroup social support [18] and greater mutual social influence [19]. It is not the groups themselves that have an impact on health and well-being, but rather it is the sense of a social identity offered by the members of the group that they share which has an effect on health and subjective well-being [20]. The literature [12] has pointed to improvement in health and psychological well-being as social identities are maintained and new ones acquired. Along these lines, we can highlight research that has revealed that maintaining the social identities people have over time as well as acquisition of acquiring new social identities improved the health of individuals suffering from depression [21,22], persons who were socially isolated [23], homeless persons [24], and individuals who had suffered a stroke [25], etc. These effects have also been examined in various different contexts. For example, a meta-analysis confirmed that social identification is positively associated with subjective well-being in organizational contexts [26].

Group memberships improve well-being because social identity increases perceived closeness with others [27] and increases access to social support [28], which provides individuals with resources to combat stress and reduce anxiety [29]. Greenaway et al. [20] hold that it is through of social identification to satisfy psychological needs (of belonging, of maintaining self-esteem, of maintaining control and the need to feel that one has a meaningful existence) [30], which brings about a positive effect on well-being and health. This is so because groups satisfy the need to belong, provide a positive social identity, are agentic by nature, and offer meaning and purpose. As a result, the health and well-being of any individual are intrinsically linked to the conditions of the group to which they form a part. Specifically, social identify can enrich and make people stronger and healthier because it provides them with self-esteem, a sense of belonging, existential sense and a feeling of control.

From the perspective of social identity, the positive effect on health and subjective well-being of participation in collective events has also been specifically analyzed [31,32]. These authors highlight association of the collective experience with activation of social identity as a central element that leads to an increase in social cohesion and its effect on the health and well-being of the participants. The concept of social identity does not only apply in the feeling of identification with the group, but also in situations in which the members of a crowd identify themselves with the same social group and with a feeling of mutual recognition as members of a common social category [33]. When individuals feel that they share the same social identify in a crowd, they behave according to the shared norms based on their social identity, see the members of the crowd as part of their group, and usually experience common collective emotions [32].

Throughout Spain, during the 99 days of the lockdown decreed by the National Government in mid-March 2020, many of its citizens went to their windows or on to their balconies at 8:00 every evening to show support for health and other professionals that were working to combat the pandemic. The groups often developed rituals, which are symbolic, repetitive and stereotypical behavior imbued with a high emotional content [34,35]. Hundreds of thousands of citizens throughout Spain publicly expressed their commitment and support for health workers fighting against the pandemic. They thereby created a feeling of unity and consensus among the population, sharing emotions that at the same time could strengthen social identity of viewing themselves as citizens of a country that is suffering from and fighting to overcome the effects of COVID-19. The expression of shared collective emotions in a group context can increase the subjective well-being of those persons who participate in acts of a group nature, since this emotional connection fosters satisfaction at finding oneself with others who share social identity as well as a feeling of collective efficacy and self-esteem [36]. It could be expected that the greater the social identification of the participants in the acts supporting the workforce fighting against the pandemic in Spain, the greater the subjective well-being experienced, in the same way that those persons who experienced a greater emotional connection in this setting also experienced greater subjective well-being. It was expected to obtain that the effect of emotional connection on subjective well-being would be positive to the extent that the collective act brings into contact normative emotions derived from the activated social identity itself.

### 1.2. Objectives and Hypotheses

The present study was aimed at analyzing if (a) the perception of social identity of individuals who participated in collective acts of support for health workers fighting against the pandemic, and (b) the emotional connection that participants experienced upon taking part in such acts, positively affected the subjective well-being of the participants, and (c) to assess if the degree of social identity plays a mediating role in the effect of emotional connection on subjective well-being. This study was carried out in May 2020, a month in which there was high infection and death rates in Spain. Every evening at 8:00 a daily act of support by citizens for health personnel fighting the COVID-19 pandemic took place. For several minutes, a large portion of Spain’s citizens went out on their balconies or to their windows to applaud the efforts of health workers. while, at the same exact time in hospitals and healthcare centers, the health workers themselves went outside their workplaces to applaud Spain’s citizens. On different city streets, ambulances, fire trucks, and police cars sounded their sirens, while people continued clapping, mixing those sounds with the sirens. Signs were also hung from balconies and windows with words of support and encouragement for health workers, and the Spanish flag and flags of different autonomous regions were displayed. This collective act took place during the 99 days that the first national lockdown in Spain lasted. All TV channels gave daily information about this act of collective support taking place in the vast majority of the Spain’s cities.

We formulated the following hypotheses:

**Hypothesis** **1.** *It is expected that those participants who score higher in social identity would feel greater subjective well-being.*

**Hypothesis** **2.** *It is expected that those participants who score higher in emotional connection would feel greater subjective well-being.*

**Hypothesis** **3.** *We propose that social identity will mediate the relationship between emotional connection and subjective well-being. This is because the effect of the emotional connection would have a positive effect on the subjective well-being of the participants to the extent that content of collective emotional expression derives from the emotional norm that the activated social identity in the collective action brings about* [32].

## 2. Materials and Methods

### 2.1. Participants and Procedure

Data collection began in mid-May 2020, in the aftermath of the state of emergency declared by the Spanish Government which facilitated a lock-down of the population and when the pandemic had already caused a high number of infections and deaths. Participating voluntarily in this study were 810 people, residents in Spain (339 women and 471 men) aged between 18 and 75 (M = 34.22; SD = 12.56), of whom 51.6% were in paid employment, 37.2% were students and 11.2% were unemployed or retired. Of those participants, 30.3% had a university degree, roughly, 72% had some type of higher education and 97.4% of the sample had Spanish nationality. The participants were mainly residents of Andalusia (31.1%), the Community of Madrid (19.4%), the Community of Catalonia (18.5%), the Community of Valencia (10.6%), the Community of Castile-La Mancha (7.1%), the Community of Castile-Leon (5.4%) and 9.2% were from other autonomous communities. The following requisites were established for participation in the study: being over 18, living in Spain during lockdown, considering oneself Spanish, participating in the acts of support for health personnel working to combat COVID-19 and signing the “informed consent”. The data were collected online by several University of Malaga psychology professors who distributed the link among the students, which in turn was shared on different social networks. Specifically, the text accompanying the survey stated: “From the University of Malaga we ask for your collaboration by answering a short survey to find out how Spanish society is facing the situation generated by the pandemic caused by COVID-19. It will not take more than 10 min of your time. Keep in mind that the survey is anonymous, and that the data will be processed as a whole and not individually. There are no right or wrong answers, so it is important that you answer honestly. Your participation is voluntary and you can withdraw from the research whenever you wish.” After giving their informed consent, the participants responded to the items. The research team was responsible for coding the data collected from each of the participants, ensuring the anonymity of the responses. The study was conducted according to the guidelines of the Declaration of Helsinki and approved by the Research Ethics Committee of the University of Malaga (Spain).

### 2.2. Instruments

Participants responded the following questionnaires:

Sociodemographic data: after signing the informed consent on the first page, and indicating regular participation in the acts of support for health personnel combatting the pandemic, the study’s participants had to indicate their gender, age, nationality, place of residence, employment situation (employed, unemployed, student, other) and education level (primary and middle school education only, further education, diploma or graduate level).

Social identity: Four items were used [37] to assess the degree to which participants felt they shared beliefs of group unity when they considered themselves citizens of the country (“we acted as a single person.” “We experienced and shared a moment of unity”, “we felt proud to belong to this group”, “we felt transformed and much more convinced of our ideas”). The response range was from 1 (not at all) to 7 (very strongly). Cronbach’s alpha coefficient was 0.89.

Emotional connection: The study used the perceived emotional synchrony short scale (PES; [38]) to assess emotional connection. This scale consisted of five items with a range of responses from 1 (not at all) to 7 (very strongly), constituting a reduced version of the scale developed by Páez et al., 2015). The items are: “we felt stronger emotions than those we feel everyday”, “we felt we were one”, “we felt a strong sense of shared emotion”, “we felt very united, almost as if we were fused together”, “we felt a very strong surge of emotion because all of us have been going through the same experience”. The participants were asked to respond to these items referring to the emotions generated by their participation in the acts of support for health personnel held daily throughout Spain. Cronbach’s alpha was 0.81.

Subjective well-being: The study used the positive and negative affects schedule, (PANAS; [39]) to measure the affective dimension of subjective well-being. The Spanish version of López-Gómez, Hervás, and Vázquez [40] was employed. It consists of 20 items, 10 of which refer to the positive affect subscale and 10 to the negative affect subscale. Each item was answered in accordance with a Likert-type rating scale with five answer options (not at all, very little, somewhat, quite a lot, very strongly). Cronbach’s alpha were 0.91 (positive affect subscale) and 0.89 (negative affect subscale). An index was obtained to measure the affective dimension of subjective well-being by subtracting the score of the negative affect subscale from the score of the positive affect subscale [41].

## 3. Results

The SPSS (version 24.0; IBM Corp., Armonk, NY, USA) was used to carry out the statistical analysis. First, we calculated joint factors of the scales social identity and perceived emotional synchrony short scale (PES) with an exploratory factor analysis. Secondly, we calculated the mean scores, standard deviations, correlations between the study’s variables, as well as differences between men and women (Student’s *t* test) for these variables. In addition, and lastly, in third place, a mediation analysis was carried out to assess to what degree social identity played a mediating role in the effect of emotional connection on subjective well-being), using the SPSS PROCESS macro [42].

Preliminary analysis. To assess the possibility of factorial commonality of the social identity and perceived emotional synchrony short scale (PES) scales, an exploratory factor analysis of principal components with varimax rotation was carried out by introducing the items of both scales into the analysis. Two factors explaining 72.20% of the variance were obtained (eigenvalue > 1). Barlett’s test of sphericity produced a chi-square statistic of 6333.40 (*p* < 0.001). The sample adequacy was acceptable, the KMO statistic being 0.859. Taking as a criterion to assign the factor with a factor loading greater than 0.50, the first factor (eigenvalue = 4.64) was saturated by the items of the social identity scale and the second factor (eigenvalue = 2.85) was saturated by the items of the PES scale.

Secondly, the data indicated that the mean scores obtained in the degree of social identity (M = 4.42; DS = 2.66) and the degree of emotional connection (M = 5.50; SD = 5.50) were scores that were higher than the mean score on the scale (Table 1). The mean score obtained in subjective well-being (obtained by subtracting the negative affect subscale scores from the positive affect subscale score) was positive (M = 0.83; SD = 1.03). Subjective well-being correlated positively with social identity (*r* = 0.21; *p* < 0.001) and with emotional connection (*r* = 0.19; *p* < 0.001). The correlation between social identity and emotional connection was positive (*r* = 0.29; *p* < 0.001). Looking at the scores obtained according to gender, the results of the *t*-test showed no significant differences in social identity (Mmen = 4.22; SD = 1.71; Mwomen = 4.51; SD = 1.62; *t* = 1.52; *p* = 0.082). Significant differences were obtained in emotional connection (Mmen = 5.20; SD = 1.30; Mwomen = 5.75; SD = 0.98; *t* = 7.70, *p* < 0.001, *d* = 1.15) and in well-being (Mmen = 1.04; SD = 1.18; Mwomen = 0.57; SD = 1.12; t = 5.77, *p* < 0.001, *d* = 1.21).

**Figure 1:**
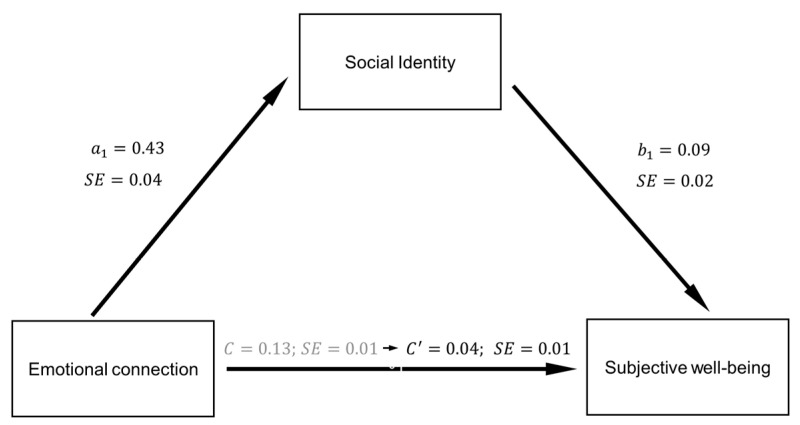
Analysis of mediation for the direct and indirect paths from emotional connection through social identity to subjective well-being. Statistically significant coefficients (*p* < 0.05).

Mediation analysis. A mediation analysis regarding the relationship between the emotional connection and subjective well-being was tested (Figure 1) using the SPSS PROCESS macro: Model 4 [42], bias-corrected 95% confidence intervals, 10,000 bootstrap samples, with emotional connection as the predictor, subjective well-being as the outcome, and social identity as mediator. Results showed that the coefficients of path *a_1_* and *b_1_* were significant, illustrating a positive association of emotional connection on social identity (*b* = 0.43, *SE* = 0.04, *t*_(810)_ = 9.90, *p* < 0.001, [0.34 to 0.51]) and a positive relationship between social identity and subjective well-being (*b* = 0.09, *SE* = 0.02, *t*_(810)_ = 2.75, *p* = 0.005, [0.07 to 0.13]; (confirming Hypothesis 1). Results had also showed the total effect (coefficient *c*) of emotional connection on subjective well-being, being a positive association (*b* = 0.13, *SE* = 0.02, *t*_(810)_ = 4.15, *p* < 0.005, [0.09 to 0.15]; (confirming Hypothesis 2). The indirect effect of emotional connection on subjective well-being (coefficient *c*) showed also a positive association (*b* = 0.04, *Boot SE* = 0.01, *BCa* 95% bias-corrected and accelerated 95% confidence interval with 10,000 samples CI = 0.03 to 0.08; (confirming Hypothesis 3).

## 4. Discussion

In the present research, we examined whether the degree of social identity among people who participated in the collective acts of support for health personnel combatting the pandemic and the degree of emotional connection positively affected subjective well-being. The results showed that the higher the participants’ degree of social identity (Hypothesis 1) and the higher degree of emotional connection (Hypothesis 2), the higher their subjective well-being scores. Furthermore, the data revealed that emotional connection had a positive effect on subjective well-being to the extent that the collective emotions generated in the collective acts of support were predetermined by the social identity activated (Hypothesis 3). The results showed a significant association between the social identity process and the collective emotions that take place in collective acts or gatherings, with social identity being a precursor to the shared emotional experience [32]. Group interaction can lead to an increase in emotional response, which, at the same time, can increase the sense of belonging, and as such, the feeling of group identity [35].

Different research undertaken from the perspective of social identity has brought to light the importance of the role played by social identity regarding its impact on health and well-being [12,16]. These studies have shown the positive and beneficial effect of identification with groups to which one belongs, as well as beneficial effects on health and well-being from participation in collective acts that enhanced and emphasized the social identity fostering participation in these collective acts [32,33]. In our research, in line with the social identity perspective, the data has shown how those individuals who have a high social identity. In line with other studies [36,37,38], our research has highlighted the effect of emotional connection on psychological well-being. Emotional connection and synchronised emotional expression (through participation in collective events widely broadcast and promoted by the country’s TV major channels) have shown that collective action can reflect and reinforce activated social identity, social cohesion and positive emotions with effects on participants’ subjective well-being [38]. The high mean scores obtained in the degree of social identity and the degree of emotional connection were obtained in the early stages of the pandemic, when participants were living that collective experience with commitment to sharing social identity as the emotional response. As has been found in other research carried out from the perspective of social identity [20], it isn’t the group itself which has an impact on subjective well-being, but rather it’s the sense of the social identity that has the impact on the subjective well-being. Collective actions that do not activate a social identity and do not provoke a synchronised emotional response should not have a positive effect on subjective well-being.

In times of the health, economic and social crisis brought about by the COVID-19 pandemic, social identity of those who participated in the collective acts on the national scale enhanced this identification with a community that was suffering from a pandemic and fighting to overcome it. These collective acts, carried out every evening by residents in Spain during the first lockdown, brought to light how everyone was affected by the same threat and was a stakeholder in the same destiny. The acts reflected their commitment and engagement with frontline health workers and their own commitment to assume responsible behavior to limit the spread of infection [6]. This type of participation in collective acts, as has been reflected in the data obtained in our study, brings about a positive relationship between social identity and an increase in subjective well-being. These types of acts, when they are mass events that successfully continue over time producing an intense emotional convergence, can lead to an increase in perception of collective efficacy and self- esteem, a sense of connectedness with others, recognition of their participation and validation of group-relevant beliefs as well as an increase in the perception of social cohesion at the community level [33,36]. Sharing the same social identity increases trust in other members of the group, as well as the desire to cooperate as a group to combat a threat that affects citizens at the personal, group, and community level [6]. The individuals who participate in such collective acts, who are aware of the presence of others that share their same social identity and who are able to feel that they are all members of that group, can feel empowered to shape their world when those around then successfully act in unison to realize shared goals [43].

This research has shown the positive effect of social identity on subjective well-being when individuals participate in an act of collective action. We can thus affirm that social participation can have these positive effects on the subjective well-being if it is able to increase the efficacy of the participants and make them feel more cohesive by interacting with others that hold similar opinions. The importance of social identity as a driving force of group and collective behavior can have important effects in times of a pandemic, such as the one we are now experiencing. Political leaders and citizens sharing the same social identity can serve to put appropriate measures into practice to limit and overcome the COVID-19 pandemic [5,6,7]. Among other factors, (a) the increase in trust and solidarity among members of the ingroup, (b) the efficacy that political figures display in their leadership, showing themselves to be a normative and prototypical exemplar of the social identity, (c) the perception that the coronavirus does not pertain to intergroup relationships (for the purpose of blaming outgroups), etc., are elements that must be taken into account to foment interventions that are capable of putting an end to the pandemic brought about by COVID-19 which has caused an enormous health and social economic crisis on a global scale. Cohesive groups sharing the same social identity are more likely to follow indications proposed by authorities to combat COVID-19. Thus, behaviors that are very important in the fight against the pandemic (e.g., admitting to being vaccinated, wearing masks, keeping a safe distance, etc.) are more likely to be followed by the population [6].

### Limitations and Future Research

This study has highlighted the positive effect of social identify and emotional connection on the subjective well-being of those persons who participated in a collective act. In our research it would have been important to take into account the degree of experience in previous participation in group events, as well as cognitive and not only affective measures of subjective well-being. It would also have been important to test the temporal effect of social identity and emotional connectedness on subjective well-being. Future research of a longitudinal nature could analyze if as individuals initiate their participation in collective acts, it provides an increase in their social identity and this then has a positive impact on subjective well-being. Future research could examine whether the positive effects of social identity and emotional connection on subjective well-being are a function of the type of collective act, the increase in collective efficacy and the subjective importance of activated social identity.

The data has shown that social identity mediates the effect of emotional connection on subjective well-being. There are studies [37] that highlight the role that collective emotional synchrony can play in the formation of group identity. From the perspective of group identity [43] it has been proposed that intra and intergroup dynamics and interaction can have effects on the evolution itself of the normative and prototypical traits of social identity that determine the behavior of the group. Future research could analyze how the synchronized group response of the group members increases the degree of social identity in acts of collective participation and their effect on subjective well-being, focusing attention not only on the intensity of the emotional response but also on the content itself of these emotions. The result of the collective act, with the level of its perceived efficacy and with possible interactions with outgroup elements, can be an influencing factor in the resulting subjective well-being.

This study has examined the positive effect of social identity and unity expressed in acts of a collective nature. Future research could focus on analyzing if a decrease in the perception of this unity and the increase in perception of the division and polarization of the group brings about negative effects on the perception of the efficacy of collective action, in the perception of group cohesion and in the subjective well-being of the individuals themselves [4]. The evolution itself of the COVID-19 pandemic in Spain with heated political clashes and fierce discrediting from the opposition towards any measure taken by the Spanish Government could be characterized by generating a highly polarized political climate, with consequences not only on the efficacy of the measures taken to combat the pandemic but even on the well-being of the population that is struggling day after day to overcome the distress caused by the health, social and economic crisis resulting from the pandemic [3].

## 5. Conclusions

From the perspective of identity, the social groups to which people belong have substantial implications for health and well-being [12]. From this perspective, it is not the groups themselves that have an impact on health and well-being, but the sense of social identity offered by the members of the group that share it which has an effect on their health and well-being [20]. There are studies that have brought to light the fact that as group identification increases, the greater the level of well-being experienced by individuals [21]. The concept and effects of social identity are also applicable in situations in which members of a crowd see themselves as members of the same group [33]. In this research, this effect has been obtained when individuals feel a heightened group identity and an elevated emotional connection when they participated in a collective act of support for healthcare workers during the first lockdown in Spain. It was precisely the participants that scored higher in group identification and emotional connection who obtained higher scores in subjective well-being. Finally, the data also revealed that emotional connection had a positive effect on subjective well-being in accordance with how the collective emotions generated in the collective acts of support were predetermined by social identity.

## Figures and Tables

**Table 1:** Descriptive statistics and Pearson correlation values between variables.

	Mean	SD	Well-Being	Social Identity	Emotional Connection
Well-being	0.83	1.03	-		
Social Identity	4.42	1.66	0.21 *	-	
Emotional Connection	5.50	1.36	0.19 *	0.29 *	-

* *p* < 0.001.

## Data Availability

The data presented in this study are available on request from the corresponding author.
